# Supplementary figures and images for: Transfer of skin microbiota between two dissimilar autologous microenvironments: A pilot study
Source: PLoS One. 2019 Dec 30;14(12):e0226857. doi: 10.1371/journal.pone.0226857 (PMC6936845; doi:10.1371/journal.pone.0226857)

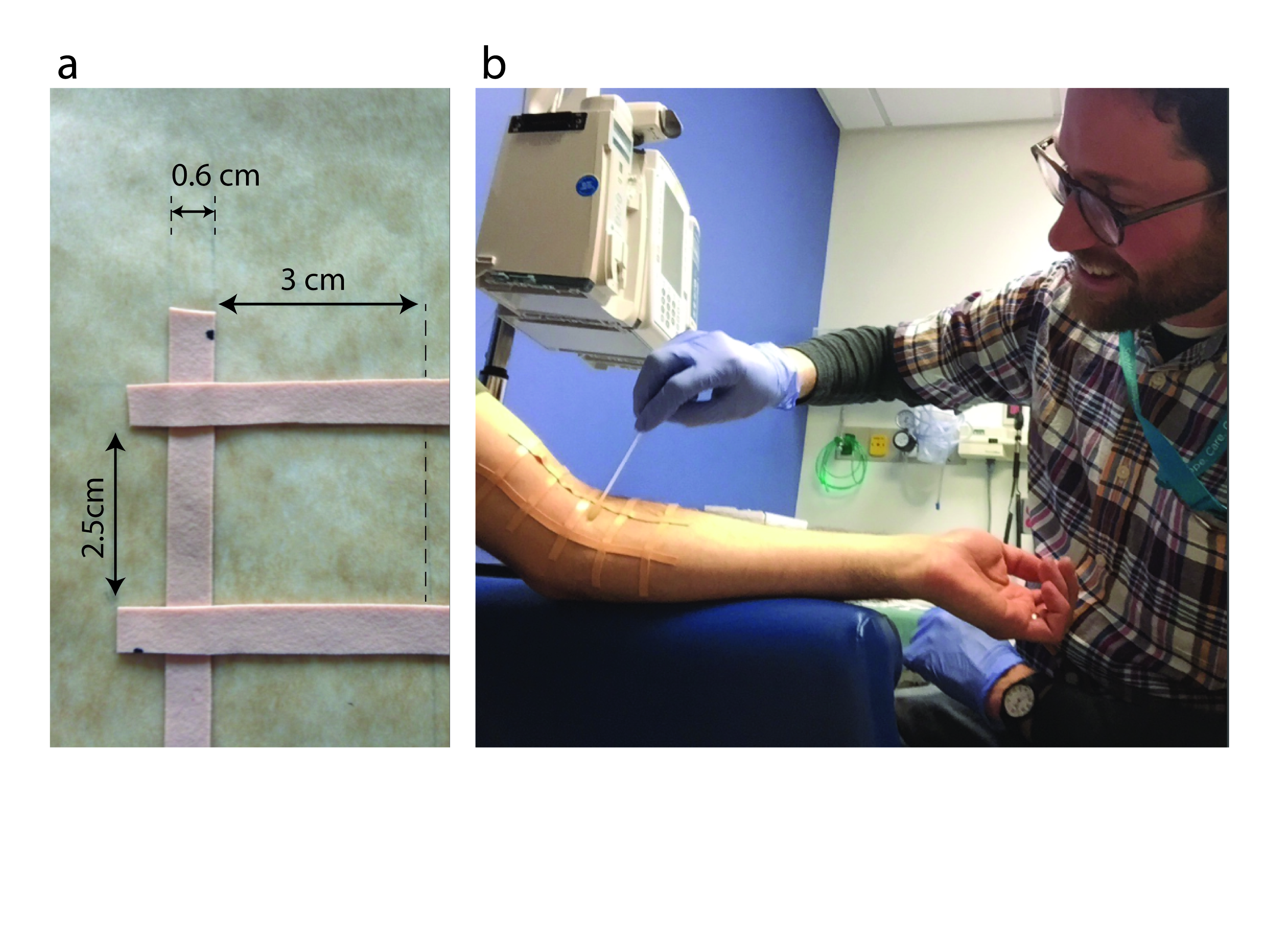

Supplement: S1 Photo — (a) we placed wax/parchment paper over a template, wiped it with bleach, and constructed the grid over it with waterproof medical tape, which had been cut into strips (~0.63cm wide, which is ¼ the width of the tape); (b) the transplant grid was easily removed like a sticker from its backing and placed on a study subject for sampling. (TIF) [file pone.0226857.s001.tif]
