# Supplementary material for: Transfer of skin microbiota between two dissimilar autologous microenvironments: A pilot study
Source: PLoS One. 2019 Dec 30;14(12):e0226857. doi: 10.1371/journal.pone.0226857 (PMC6936845; doi:10.1371/journal.pone.0226857)
Supplement: Attachment — Submitted filename: Response to Reviewers.docx [file pone.0226857.s003.docx]

11/3/19

Benji Perin, MD

University of Washington Division of Dermatology

Seattle, WA

**Response to reviewers**, for **"**Transfer of skin microbiota between two dissimilar autologous microenvironments: a pilot study",

To the PLOS ONE editorial staff and reviewers,

Many thanks for your thoughtful review of our manuscript. We have made every attempt to revise our paper with care, incorporating your comments and criticisms, or explaining why we have not made changes in a few select cases.

Sincerely,

Benji Perin, MD

University of Washington

*Reviewer #1: The primary concern is a lack of agreeable definition for “transfer”. In this space the authors have assumed that the detection of DNA signature 0 or 24 hour after swabs constitutes transfer. As written, there is a possibility that they only transferred DNA, not viable organisms. In fact, the culture results in Table 3 argue strongly that they did not transfer a “viable community”, but at best transferred two coagulase negative Staph strains. Traditional culture techniques should not miss Staph epi – thus the lack of S. epi growth* *in the T24 cultures is concerning that no viable organisms were transferred in the first place.*

We appreciate the reviewer's clear criticism of our language regarding "transfer", and we agree that we must qualify our conclusions and acknowledge only what we accomplished (transfer of partial DNA signature). A strict interpretation of our data (which is the only one worthwhile) offers only limited evidence that this DNA was from live, transferred bacteria. Despite living with this data for a few years now and thinking through several versions of the manuscript, it wasn't until really working through this reviewer's comments (especially with the guidance on controls) that I realized how much more guarded we have to be with regards to our conclusions. This realization has helped us with revision at many places in the manuscript.

I do see in the comment above that we did not clearly articulate what we accomplished with our robust culture results: "*Traditional culture techniques should not miss Staph epi – thus the lack of S. epi growth* *in the T24 cultures is concerning that no viable organisms were transferred in the first place"*. While our cultures did not capture the rare organisms unique to the arm (either in baseline from the arm or in the transplant), our cultures yielded >900 distinct colonies, most of which were identified by MALDI-TOF. In particular, these data show that T_24_ cultures yielded considerable quantities of all the typical skin flora that one would expect including lots of *Staphylococcus epidermidis*. I believe the reviewer is noting that we did not see the particular morphotype of *S. epi* persist at T_24_ that we identified as having transferred at T_0_, despite the fact that the T_24_ media plates were busy with many species of *Staphylococcus*, including distinct morphotypes of *S. epi*. We are including this culture data as a supplementary file in the revision.

*A control could be performed swabbing the back with heat killed samples from the arm (i.e. culture Staph from the arm, heat kill it, then see if the bacterial 16S signature is present on the T0 and T24 samples). If the non-viable swab shows a less impressive transfer, the authors would have a much stronger claim. IE consider the direct non-viable culture swab a DNA-only, positive control to compare. An alternate approach would be to take the T0 swab and dip it in diluted EtOH prior to swabbing the back. If the 16S signature differed between the T24 and T24+EtOH group (versus a blank swab dipped in EtOH alone) the authors might have a stronger case that the T24 signature was from viable organisms.*

We very much appreciate this suggestion. Retrospectively I don't see how we missed this for so long - perhaps because we were focused on what we thought we could achieve (or thought we did achieve) by culture. We have incorporated this suggestion into our limitations and future directions section.

*Short of control samples, the entire paper (especially the abstract) requires that the language be amended to outline that they have transferred the DNA profile of the arm to the back. They have not provided the results needed to claim that they “can move viable skin organisms from one site to another”. There is no shame in showing that they were the first to transfer a partial DNA signature, but need to do further work to establish if they transferred viable organisms. But this paper claims multiple times to have transferred viable communities when they do not demonstrate that with their data.*

We have addressed this point throughout the Results and Discussion, in addition to the Abstract.

*This also needs to be discussed in detail in the limitations section, rather than the glancing comment contained now. As constructed, I feel that the overall language is misleading as it implies that swabs could be used from person A to person B to transfer viable organisms. This may be true, and is indeed “worthy of further investigation”, but without demonstrating colonization (even for as little as 24 hours), I cannot endorse this manuscript.*

We have approached this comment head on in our limitations section in addition to other parts of the discussion (in addition to the abstract).

*Minor concern:
In Fig 5, the authors state that the orange symbols – “the community has shifted towards the Ba samples (red) and away from the Bb samples (yellow)”. I don’t see this claim supported at all, can they provide mathematical support for this?*

I clarified what we were attempting to show with this figure in the results section and in the caption for the figure. The basis for our interpretation is qualitative - if the new context we provide for this figure is not sufficient, it may have to be deleted.

*Reviewer #2: Perin et al present skin microbiome transplantation auto-transplantation from a site on the back to an arm site study in four patients. This work results interesting for the community but some aspects need to be clarified.*

*Major comments:
-It results unclear how it was measured the viability of the applied organisms on the transplant. It should be clarified how many live bacteria were transferred. Viability of the isolated microorganisms should be assayed and quantified*

We have made every attempt to clarify what conclusions about viability can be drawn from our results. (Please see above and revision to Abstract, Results, and Discussion sections)

*-Figures 3 and 5 should include the data of T24*

For figure 3, I have added the line: "There was no significant difference nor trend comparing Bb_24_ and T_24_ samples, for this reason we have not included them here." For figure 5 the graphic became very busy with the incorporation of the Bb_24_ and T_24_ data points, and showed no clear differences in community composition (just like in Fig 3). For this reason we focused on the T_0_ timepoint.

*Minor comments:
-What is the composition of the saline solution used for recovery?*

We have added this line to the paper: To assess bioburden, a moistened swab (BD, ESwab) with 0.85% sterile saline; (Remel) was vigorously rubbed on a 2cm x 2 cm area of antecubital fossa. This is the same saline we use throughout the experiment, including for collection of baseline samples, collecting bacteria for transfer pellet, and recovering the transferred pellet.

*-Tables are very hard to read. Version with improved resolution should be provided.*

The tables have been incorporated into the text of the manuscript.

**In addition to the changes made to the manuscript based on the reviewers' comments above, we have**:

- made changes to file names and formatting of the manuscript (heading formatting, title page formatting, figure legend formatting, tables, citations)

- added statement that we obtained written informed consent from participants in methods section, obtained consent for the identifiable individual in the supplementary photo

- included separate caption for each figure in manuscript

- included tables as part of main manuscript and removed individual files.

- included captions for Supporting Information files at the end manuscript
